# Method of contraception and risk of ovarian cancer data

**DOI:** 10.1016/j.dib.2021.107469

**Published:** 2021-10-10

**Authors:** Jacqueline Chesang, Ann Richardson, John Potter, Mary Sneyd, Pat Coope

**Affiliations:** a School of Health Sciences, University of Canterbury, Christchurch, New Zealand; b Centre for Public Health Research, Massey University, Wellington, New Zealand; c Hugh Adam Cancer Epidemiology Unit, Preventive and Social Medicine, University of Otago, New Zealand; d College of Education, Health and Human Development, University of Canterbury, New Zealand; e School of Public Health, University of Nairobi, Kenya

**Keywords:** Ovarian cancer, DMPA, Vasectomy, IUDs, Tubal ligation, Contraceptives

## Abstract

The data presented here were obtained for a New Zealand nationwide population-based case-control analysis undertaken to assess the association between ovarian cancer and depot medroxyprogesterone acetate (DMPA), intrauterine contraceptive devices (IUDs), and vasectomy of a woman's sexual partner (Chesang et al., 2021). The research involved women aged 35 to 69 years. Controls were randomly selected from the New Zealand electoral roll. Cases were women with a diagnosis of incident ovarian cancer recruited from the New Zealand Cancer Registry and had to be listed on the electoral roll.

Data collection was conducted between 1st May 2013 and 31st October, 2015. A structured postal questionnaire was used to gather information. Data were analysed using IBM Statistical Package for the Social Sciences (IBM SPSS statistics 22). Odds ratios adjusted for age were calculated using the method of Mantel and Haensze (Rosner et al., 2007). For multivariable analyses, binary logistic regression was used.

Description of study participants and age-adjusted and multivariable analyses of the association between ever-use and specifics of use of DMPA, IUDs, and vasectomy were presented in a journal article (Chesang et al., 2021). Here, we present data from analyses of the risk of ovarian cancer by histological type associated with the use of DMPA, IUDs and ever having had a vasectomised partner. In addition, analyses assessing the association between ovarian cancer and these contraceptives restricted to ever-users and never-users of hormonal contraceptives (defined as oral contraceptives or DMPA) are presented. Data from analyses of the association between history of tubal ligation and the risk of ovarian cancer are also presented.

These data, including the findings of a related study (Chesang et al., 2021) and the raw data, can be included in a collaborative analysis of existing studies undertaken to assess the association between IUDs, long-acting progestogen-based contraceptives, and partner vasectomy and the risk of ovarian cancer.

## Specifications Table

**Table:** 

Subject	Health and Medical sciences
Specific subject area	The data presented are on the epidemiology of ovarian cancer, specifically, the association between ovarian cancer and use of different contraceptive methods.
Type of data	Table
How data were acquired	Data were collected using a structured questionnaire. (The questionnaire is submitted as supplementary material).
Data format	Analyzed. (The raw data is submitted as supplementary material).
Parameters for data collection	Participants were asked about ever-use, age at first use, time since last use, and duration of use of OCs, DMPA, IUDs and contraceptive implants. Information on type(s) of IUD(s) used, history of and age at TL, duration of reliance on condoms, partner vasectomy, or ‘other’ contraceptives, socio-demographic characteristics of participants, menstrual and reproductive history, family history of cancer, and potential confounding factors were sought.
Description of data collection	Each potential participant was sent a letter accompanied by an information and consent form, a copy of the questionnaire, and a post-paid addressed envelope for returning the questionnaire and signed consent form. Women who did not respond to the initial questionnaire within three weeks from the date of dispatch were sent a second data collection pack. Women who did not respond to the second mail-out were contacted and asked to complete the questionnaire by telephone. If they were willing to do this, a telephone interview was carried out using the same questionnaire. All questionnaires were checked for completeness and, where necessary, participants were contacted to obtain missing data.
Data source location	Country: New Zealand
Data accessibility	With the article.
Related research article	J. J. Chesang, A. K. Richardson, J. D. Potter, M. J. Sneyd and P.Coope, Association of partner vasectomy, depot medroxyprogesterone acetate and intrauterine contraceptive devices with ovarian cancer, Annals of Epidemiology. 60 (2021) 15-20. https://doi.org/10.1016/j.annepidem.2021.04.006

## Value of the Data

• Knowledge of the effects of contraceptives on the health of users is important in assessing the risks and benefits of a contraceptive when choosing a method. Contraceptives are widely used and use may span months or years; therefore, even a small effect on the risk of ovarian cancer may have a great impact on the incidence of disease in the general population.
• The inverse associations of both partner vasectomy and use of DMPA with the occurrence of ovarian cancer provide additional important non-contraceptive benefits of vasectomy and DMPA. This may provide alternative contraceptive options for women who wish to lower their risk of ovarian cancer but cannot use oral contraceptives.
• These data, including the findings of a related study [1] and the raw data, can be included in a collaborative analysis of existing studies undertaken to assess the association between IUDs, long-acting progestogen-based contraceptives, and partner vasectomy and the risk of ovarian cancer.

## 1 Data Description

Description of research participants and age-adjusted and multivariable analyses of the association between ever-use and specifics of use of depot medroxyprogesterone acetate (DMPA), intrauterine contraceptive devices (IUDs), and vasectomy were presented in an article [1]. Here, we present supplementary analyses of the association between use of these contraceptives and the risk of ovarian cancer. Analyses of the association between history of tubal ligation (TL) and the risk of ovarian cancer are also presented. This section ends with a description of the raw data.

### 1.1 Risk of ovarian cancer associated with ever having had a vasectomised partner, ever-use of DMPA and ever-use of IUDs by histologic type

Analysis of risk by histological type of ovarian cancer associated with ever-use of DMPA, IUDs and vasectomy was performed (Table 1). Due to the small numbers, cases were categorised as serous and non-serous, and mucinous and non-mucinous. Risk for serous and non-serous tumours was assessed in relation to ever-use of DMPA, IUDs and vasectomy, adjusted for age in 5-year groups. Risk for mucinous and non-mucinous tumours was also assessed, adjusted for age (categorised into <60 years and ≥60 years due to the small number of cases with mucinous tumours). The numbers were small for multivariable analysis and for assessment by other histologic types.

**Table 1:** Risk of ovarian cancer associated with ever having had a vasectomised partner, ever-use of DMPA and ever-use of IUDs by histological type.

	Cases		Controls			95% CI		
	*No.*	*(%)*^1^	*No.*	*(%)*^1^	OR	*Lower*	*Upper*	*P*-value
	*Serous*^2^							
Vasectomy Use	*(n=90)*		*(n=744)*					
Never	53	(59)	412	(55)	*1.00*			
Ever	37	(41)	332	(45)	0.83	0.53	1.31	0.495
DMPA Use	*(n=90)*		*(n=745)*					
Never	83	(92)	657	(88)	*1.00*			
Ever	7	(8)	88	(12)	0.70	0.31	1.58	0.502
IUD Use	*(n=90)*		*(n=745)*					
Never	65	(72)	554	(74)	*1.00*			
Ever	25	(28)	191	(26)	1.04	0.63	1.72	0.979
	*Non-serous*^2^							
Vasectomy Use	*(n=52)*		*(n=744)*					
Never	39	(75)	412	(55)	*1.00*			
Ever	13	(25)	332	(45)	0.41	0.22	0.79	0.009
DMPA Use	*(n=52)*		*(n=745)*					
Never	47	(90)	657	(88)	*1.00*			
Ever	5	(10)	88	(12)	0.70	0.27	1.83	0.614
IUD Use	*(n=52)*		*(n=745)*					
Never	40	(77)	554	(74)	*1.00*			
Ever	12	(23)	191	(26)	0.86	0.44	1.66	0.767
	*Mucinous*^3^							
Vasectomy Use	*(n=10)*		*(n=744)*					
Never	7	(70)	412	(55)	*1.00*			
Ever	3	(30)	332	(45)	0.53	0.14	2.07	0.546
DMPA Use	*(n=10)*		*(n=745)*					
Never	8	(80)	657	(88)	*1.00*			
Ever	2	(20)	88	(12)	1.89	0.39	9.18	0.763
IUD Use	*(n=10)*		*(n=745)*					
Never	9	(90)	554	(74)	*1.00*			
Ever	1	(10)	191	(26)	0.32	0.04	2.56	0.446
	*Non-Mucinous*^3^							
Vasectomy Use	*(n=132)*		*(n=744)*					
Never	85	(64)	412	(55)	*1.00*			
Ever	47	(36)	332	(45)	0.69	0.47	1.01	0.073
DMPA Use	*(n=132)*		*(n=745)*					
Never	122	(92)	657	(88)	*1.00*			
Ever	10	(8)	88	(12)	0.60	0.30	1.19	0.185
IUD Use	*(n=132)*		*(n=745)*					
Never	96	(73)	554	(74)	*1.00*			
Ever	36	(27)	191	(26)	1.08	0.71	1.64	0.801

1 Percentages are of total stated

2 ORs adjusted for age in five-year groups

3 ORs adjusted for age categorised into <60 and ≥60 years due to the small number of cases with mucinous tumours.

### 1.2 Risk of ovarian cancer associated with ever having had a vasectomised partner and ever-use of IUDs, restricted to ever- and never-users of hormonal contraceptives (DMPA or OCs)

The association between risk of ovarian cancer and partner vasectomy or use of IUDs was assessed confined to women who had ever-used hormonal contraceptives (defined as oral contraceptives [OCs] or DMPA) (Table 2). Among women who had ever-used hormonal contraceptives, risk of ovarian cancer among women who have ever had a vasectomised partner and those who have ever-used IUDs was compared to the risk in those who have never had a vasectomised partner and those who have never-used IUDs, respectively.

**Table 2:** Risk of ovarian cancer associated with ever having had a vasectomised partner, and use of IUDs in ever- and never-users of hormonal contraceptives (DMPA or OCs).

	Cases		Controls			95% CI				95% CI		
	*No.*	*(%)*^3^	*No.*	*(%)*^3^	OR^1^	*Lower*	*Upper*	P-Value	OR^2^	*Lower*	*Upper*	P-Value
Ever-users of hormonal contraceptives (DMPA/OCs)												

Vasectomy	*(n=118)*		*(n=674)*									
Never	68	(58)	358	(53)	*1.00*				*1.00*			
Ever	50	(42)	316	(47)	0.81	0.54	1.21	0.355	0.84	0.54	1.28	0.410
IUD Use	*(n=118)*		*(n=675)*									
Never	83	(70)	495	(73)	*1.00*				*1.00*			
Ever	35	(30)	180	(27)	1.09	0.71	1.68	0.772	1.14	0.72	1.81	0.572

Never used hormonal contraceptives (DMPA/OCs)												

Vasectomy Use	*(n=34)*		*(n=70)*									
Never	30	(88)	54	(77)	*1.00*				*1.00*			
Ever	4	(12)	16	(23)	0.53	0.17	1.71	0.407	0.52	0.11	2.53	0.244
IUD Use	*(n=34)*		*(n=70)*									
Never	29	(85)	59	(84)	*1.00*				*1.00*			
Ever	5	(15)	11	(16)	1.03	0.31	3.41	0.799	3.33	0.61	18.32	0.166

1 Adjusted for age in five-year groups

2 Adjusted for age in 5-year groups; PMH (ever-use); OCs (ever-use); Parity (grouped into 1, 2, 3, and ≥4); history of ovarian, breast, endometrial, or colorectal cancer in a first-degree relative; age at last delivery (grouped into: ≤25, 26-30, 31-35, and >35); history of infertility.

3 Percentages are of total stated

Analyses restricted to never-users of hormonal contraceptives were also performed (Table 2, bottom panel). There were 4 cases and 16 controls with a history of union with a vasectomised partner, who had never used hormonal contraceptives. These were compared to women who had never used hormonal contraceptives and had never had a vasectomised partner. Among ever-users of IUDs, 5 cases and 11 controls had never used OCs or DMPA. These were compared to women who had neither used hormonal contraceptives nor IUDs.

### 1.3 Analysis of the risk of ovarian cancer associated with ever-use of DMPA restricted to ever- and never-users of OCs

In Table 3 are analyses of the association between ever-use of DMPA and the risk of ovarian cancer confined to ever- and never-users of OCs. For the two groups of women (those who have ever and those who have never-used OCs), the risk of ovarian cancer among women who have ever-used DMPA was compared to that among women who have never-used DMPA. For these restricted analyses, the numbers were too small to allow for assessment of specifics of use.

**Table 3:** Risk of ovarian cancer in ever-users of DMPA compared to never-users in women who have ever-used oral contraceptives and in those who have never-used oral contraceptives.

	Cases		Controls			95% CI				95% CI		
	*No.*	*(%)*^3^	*No.*	*(%)*^3^	OR^1^	*Lower*	*Upper*	*P*-Value	OR^2^	*Lower*	*Upper*	*P*-Value
Ever-users of oral contraceptives												

DMPA Use	*(n=116)*		*(n=668)*									
Never	105	(91)	587	(88)	*1.00*				*1.00*			
Ever	11	(9)	81	(12)	0.78	0.40	1.52	0.569	0.74	0.36	1.52	0.412

Never-users of oral contraceptives												

DMPA Use	*(n=36)*		*(n=77)*									
Never	34	(94)	70	(91)	*1.00*				*1.00*			
Ever	2	(6)	7	(9)	0.42	0.07	2.52	0.591	0.39	0.06	2.63	0.332

1 Adjusted for age in five-year groups

2 Adjusted for age in 5-year groups; PMH (ever-use); OCs (ever-use); Parity (grouped into 1, 2, 3, and ≥4); history of ovarian, breast, endometrial, or colorectal cancer in a first-degree relative; age at last delivery (grouped into: ≤25, 26-30, 31-35, and >35); history of infertility.

3 Percentages are of total stated.

### 1.4 Risk of ovarian cancer in women with history of tubal ligation

Analyses assessing the risk of ovarian cancer in women with history of TL compared to those with no history of TL are presented in Table 4. Risk of ovarian cancer in relation to age at and time (in years) since TL are also presented. Only one control had had a TL reversal operation with the delivery of one child post-reversal and, therefore, the effect of reversal on the risk of ovarian cancer could not be assessed. This participant was excluded from the analyses.

**Table 4:** Risk of ovarian cancer in women with history of tubal ligation.

	Cases		Controls			95% CI		
	*No.*	*(%)*^2^	*No.*	*(%)*^2^	OR^1^	*Upper*	*Lower*	*P*-value
Tubal Ligation								
No	121	(82)	600	(80)	1.00			
Yes	27	(18)	146	(20)	0.98	0.62	1.57	0.963
Age at TL								
Never had TL	121	(82)	600	(80)	1.00			
≤30 (22-30)	10	(7)	57	(8)	0.94	0.46	1.92	0.999
31-35	8	(5)	46	(6)	0.92	0.42	2.02	0.997
36-40	7	(5)	26	(3)	1.37	0.58	3.28	0.638
>40 (41-56)	2	(1)	17	(2)	0.581	0.13	2.54	0.673
*Trend Test- per year*					1.001	0.929	1.079	0.980
Years Since TL								
Never had TL	121	(82)	600	(80)	1.00			
≤10 (1-10)	2	(1)	10	(1)	1.01	0.21	4.76	0.697
11-20	6	(4)	18	(2)	1.40	0.53	3.66	0.675
21-30	9	(6)	54	(7)	0.88	0.42	1.86	0.877
>30 (31-45)	10	(7)	64	(9)	0.91	0.44	1.91	0.953
*Trend Test – per year*					*0.994*	*0.925*	*1.067*	*0.860*

1 Adjusted for age in five-year groups

2 Percentages are of total stated

Analyses restricted to women who had ever-used, and those who had never-used, hormonal contraceptives were also done (Table 5). In these two groups (ever- and never-users of hormonal contraceptives) the risk of ovarian cancer among women who have had TL was compared to that in those who have not had TL.

**Table 5:** Association between tubal ligation and the risk of ovarian cancer in ever-users and never-users of DMPA and/or oral contraceptives.

	Cases		Controls			95% CI		
	*No.*	*(%)*^2^	*No.*	*(%)*^2^	OR^1^	*Lower*	*Upper*	*P*-Value
Ever-used hormonal contraceptives								

Ever had TL								
No	91	(79)	543	(80)	1.00			
Yes	24	(21)	134	(20)	1.14	0.69	1.90	0.705

Never-used hormonal contraceptives								

Tubal Ligation								
No	30	(91)	57	(83)	1.00			
Yes	3	(9)	12	(17)	0.61	0.16	2.40	0.700

1 Adjusted for age in five-year groups

2 Percentages are of total stated

### 1.5 Raw data

The raw data (submitted as a supplementary file) contains all the data collected for this research, including for the published article [1]. The data are coded and the interpretation of the codes is provided on a separate tab. Where calculations were done, these have been described. The questionnaire used to gather information is also submitted as a supplementary file.

## 2 Experimental Design, Materials and Methods

The data presented here are from a national population-based case-control study conducted in New Zealand. Approval to conduct the study was obtained from the Southern Health and Disability Ethics Committee (13/STH/26) and the University of Canterbury Human Ethics Committee (HEC2013/08). Recruitment of participants and data collection were done nationwide. Data were collected from May 2013 through October 2015.

Cases were women with newly diagnosed, histologically confirmed ovarian cancer, aged 35 to 69 years, at any stage of disease but capable of effective communication in English. The controls consisted of women free from ovarian cancer with similar age restrictions and capable of effective communication in English. Ovarian cancer is generally a disease of post-menopausal women, with the highest incidence at ages 65 to 74 years. Women use contraceptives during their reproductive years; that is, between menarche and menopause. The age range, 35 to 69 years inclusive, captures the population that is most affected by ovarian cancer and, at the same time allows for best recall of contraceptive usage. Due to logistical constraints, potential participants who were unable to communicate in English were excluded. English being the most spoken language in New Zealand and participants being New Zealand citizens or permanent residents, this requirement was not expected to exclude a significant number of potential participants. (For the control arm, of 1,903 potential participants approached, only 16 could not communicate in English.)

Controls were randomly selected from the General and Māori Electoral Rolls, frequency matched to cases by 5-year age groups. Access to electronic data about people on the New Zealand electoral roll for purposes of health research is allowed under Section 112(3) of the Electoral Act 1993. Cases were recruited sequentially from the New Zealand Cancer Registry (NZCR) and were eligible if they were also listed on the electoral rolls. Histology reports for all women with a recent diagnosis of ovarian cancer were forwarded to the research team from the NZCR (in accord with legislated provision for access to Cancer Registry data for health research). The reports were reviewed by a medically qualified member of the research team. All women who met the inclusion criteria for the study were selected. Approval to approach the patients about the study was then sought from each woman's relevant doctor. In NZ, there are statutory requirements for laboratories to report all cancer diagnoses (except non-melanoma skin cancer) to NZCR and for all New Zealand citizens and permanent residents 18 years and above to register on the parliamentary electoral roll. To ensure that the controls represented the population from which the cases came, and that cases and controls could be contacted by mail, all eligible cases and controls were listed on the electoral roll. Participants were stratum-matched by age because the incidence of ovarian cancer increases with age. Approval from the doctors of the women who constitute the cases was sought with the assumption that each doctor knew the woman well and would, therefore, know whether she was well enough to participate in the study. In view of the small number of ovarian cancer cases, more controls than cases were recruited (a case-control ratio of 1:5) in order to enhance the statistical power of the study.

The data were collected using a structured questionnaire that included previously validated questions (from the New Zealand census for demographic and other relevant information) and questions sourced from suitable questionnaires such as those used in the Million Women Study [3]. Information sought included socio-demographic characteristics (age, area of residence, country of birth, ethnicity, occupation, level of income, and level of education), history of contraceptive use (types of contraceptives used, age at first use, time since last use and duration of use), menstrual and reproductive history (ages at menarche and at menopause, menopausal status, average menstrual cycle length and regularity, parity, and duration of breast feeding), family history of cancer (type of cancer, relationship of the affected family member to the participant and age at diagnosis), and other protective and risk factors for ovarian cancer. The histopathology report for each case provided by the NZCR included date of birth, date of diagnosis, histologic type of cancer, stage and grade of disease at diagnosis, and name of the treating doctor.

Each potential participant was sent a letter on University of Canterbury letterhead, signed by two members of the research team. The letter was accompanied by an information and consent form, a copy of the questionnaire, and a postage-paid addressed envelope for returning the questionnaire and signed consent form. Participants were provided with a calendar of major life events, as part of the questionnaire, to assist in recall and to record their use of contraceptives. To facilitate responses, women who did not respond to the initial questionnaire and consent form within three weeks from the date of dispatch, were sent a second letter, information and consent form, questionnaire, and postage-paid addressed envelope. Women who did not respond to the second mail-out were contacted and asked to complete the questionnaire by telephone interview conducted by a trained interviewer. If they were willing to do this, a telephone interview was done, using the same questionnaire. They were requested to post back a signed consent form. Those who returned completed questionnaires without a signed consent form or who had a telephone interview and did not post back a signed consent form were considered to have consented. All questionnaires were checked for completeness; where necessary, participants were contacted to obtain missing data and to clarify discrepant values.

Data were entered into an EXCEL spreadsheet. Frequency tables were constructed in order to aid in identifying data entry errors. These errors were corrected by referring to the answers in the questionnaires. In situations where values provided were considered unrealistic, these were treated as missing values. All identifying information was coded before analysis. To ensure that participants were at risk of a first primary ovarian cancer, women with a prior history of ovarian cancer were excluded. Controls with history of bilateral oophorectomy were also excluded because this effectively eliminates the risk of developing ovarian cancer.

Data were analysed using the IBM Statistical Package for the Social Sciences (IBM SPSS statistics 22). Descriptive statistics were used to compute frequencies and percentages. All variables collected in the study were assessed for association with the risk of ovarian cancer, adjusted for age in 5-year groups using the method of Mantel and Haenszel [2]. When controlling for more than one variable, binary logistic regression was used. Odds ratios, 95% confidence intervals, and p-values were reported. All statistical tests were 2-sided and the risk estimates were considered statistically significant if the p-value was less than 0.05. Tests for trend were done using continuous rather than categorical variables. Ever-use of IUDs, vasectomy, and DMPA were also adjusted for ever-use of OCs, ever-use of post-menopausal hormone, history of ovarian, breast, endometrial, or colorectal cancer in a first-degree relative, age at last delivery, and age in 5-year groups. These factors were selected based on prior literature indicating their association with ovarian cancer. In addition, they had the strongest association with the risk of ovarian cancer and had a statistically significant independent association when entered together into the logistic regression model. Reference age was defined as age at diagnosis for cases and age at participation (completion of questionnaires) for controls. (The average duration from diagnosis to questionnaire completion for cases was 5.1 months, standard deviation 2.4. Analyses with 5.1 months subtracted from the control ages did not change the results, so the reference ages were used in the analyses.)

In addition, analyses of the risk of ovarian cancer associated with ever having had a vasectomised partner, history of TL, and use of DMPA and IUDs restricted to women who have ever-used and those who have never used hormonal contraceptives (defined as OCs or DMPA) was performed.

## Ethics Statement

Approval to conduct the study was obtained from the Southern Health and Disability Ethics Committee (13/STH/26) and the University of Canterbury Human Ethics Committee (HEC 2013/08). Approval to approach the patients (cases) was sought from each woman's doctor. All participants gave informed consent to participate in the study.

## CRediT authorship contribution statement

**Jacqueline Chesang:** Investigation, Data curation, Formal analysis, Writing – original draft. **Ann Richardson:** Investigation, Data curation, Formal analysis, Writing – original draft. **John Potter:** Investigation, Formal analysis. **Mary Sneyd:** Investigation. **Pat Coope:** Formal analysis.

## Declaration of Competing Interest

The authors declare that they have no known competing financial interests or personal relationships which have or could be perceived to have influenced the work reported in this article.

This work was supported by Genesis Oncology Trust (E6083) and Wayne Francis Charitable Trust (GOT-1262-RPG). However, Genesis Oncology Trust and Wayne Francis Charitable Trust were not involved in the design of the study and collection, analysis and interpretation of data and in writing the manuscript.

## Appendix Supplementary materials

Image, application 1

Image, application 2

Image, application 3

Image, application 4

Image, application 5
