# Supplementary material for: Method of contraception and risk of ovarian cancer data
Source: Data Brief. 2021 Oct 10;39:107469. doi: 10.1016/j.dib.2021.107469 (PMC8640863; doi:10.1016/j.dib.2021.107469)
Supplement: Supplementary file 1 [file mmc1.docx]

## APPENDIX 7: QUESTIONNAIRE

**School of Health Sciences**

Tel: +64 3 366 7001 ext. 8691

www.health.canterbury.ac.nz

healthsciences@canterbury.ac.nz
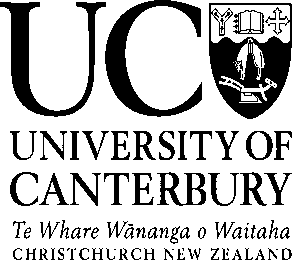


**CANCER AND WOMEN’S REPRODUCTIVE HEALTH STUDY**

***Please follow the following guidelines in filling the questionnaire:***

- *Please clearly mark your answers on the appropriate box/boxes.*
- *Put numbers in the appropriate boxes, for example 15^th^ May 1995*

| ***1*** | ***5*** | */* | ***0*** | ***5*** | */* | ***1*** | ***9*** | ***9*** | ***5*** |
| --- | --- | --- | --- | --- | --- | --- | --- | --- | --- |

- *Print your answers in capital letters in the spaces provided.*
- *Please answer every question as completely as possible.*
- *If you cannot remember the exact date or age, please give an approximate answer.*

| Cancer and Women’s Reproductive Health Study, Version 2 1^st^ December, 2013 Page \| 1 |
| --- |

**SECTION A: GENERAL QUESTIONS ABOUT YOU**

1. When were you born? *(Please put day/month/year)*

//

1. How old are you?  years
2. Where do you usually live?

Suburb or rural locality

City, town or district

Country

1. Which country were you born in?

New Zealand

Australia

England

Scotland

China (People’s Republic of)

India

South Africa

Samoa

Cook Islands

Other *(Please print the present name of the country*)

| Cancer and Women’s Reproductive Health Study, Version 2 1^st^ December, 2013 Page \| 2 |
| --- |

1. If you live in New Zealand but you were **not** born here, answer this question.

When did you first arrive to live in New Zealand?

Month if known Year

(e.g 03) (e.g 1990)

1. Which ethnic group do you belong to?

*(Mark the space or spaces which apply to you*)

New Zealand European

Māori

Samoan

Cook Island Māori

Tongan

Niuean

Chinese

Indian

Other such as DUTCH, JAPANESE, TOKELAUAN. Please state:

| Cancer and Women’s Reproductive Health Study, Version 2 1^st^ December, 2013 Page \| 3 |
| --- |

**SECTION B: QUESTIONS ABOUT YOUR USE OF CONTRACEPTIVES (FAMILY PLANNING METHODS)**

*To answer this section, use the calendar of life events provided to aid you in recall****.*** *Please indicate all the contraceptive methods you have ever used*

**CALENDAR OF MAJOR LIFE EVENTS**

| **LIFE EVENT** | **Year the event occurred** | **How old were you when the event occurred?** | **Type of contraceptive method/s (family planning method/s) you used after the event.** |
| --- | --- | --- | --- |
| The first menstrual period in your life |  |  |  |
| The first time you had sex |  |  |  |
| Birth of 1^st^ child |  |  |  |
| Birth of 2^nd^ child |  |  |  |
| Birth of 3^rd^ child |  |  |  |
| Birth of 4^th^ child |  |  |  |
| Birth of 5^th^ child |  |  |  |
| Birth of 6^th^ child |  |  |  |
| Birth of 7^th^ child |  |  |  |
| Birth of 8^th^ child |  |  |  |
| Birth of 9^th^ child |  |  |  |
| Birth of 10^th^ child |  |  |  |
| Menopause (the last menstrual period in your life) |  |  | ***What contraceptive method were you on around the time of your menopause?*** |

| Cancer and Women’s Reproductive Health Study, Version 2 1^st^ December, 2013 Page \| 4 |
| --- |

1. Have you ever used oral contraceptive pills? *(this includes ‘the pill’ and the mini-pill)*

No *If* ***No,*** *please go to question 10*

Yes

If **YES**:

-about how old were you when you first went on the pill?  years.

-are you still on the pill?  No, stopped – If so, when?  years ago

Yes, still using

-for how many years in total did you take the pill?  years

*(Add together the years and months when you actually took the pill.*

*Please write “0” if you used the pill for less than a year in total)*

1. Have you ever used oral contraceptive for any other purpose other than as a family planning method? *(e.g. endometriosis, acne etc)*

No *If* ***No,*** *please go to question 10*

Yes

If **YES**, please state the reason(s) for using it

………………………………………………………………………………….

…………………………………………………………………………………..

…………………………………………………………………………………..

For how many years in total did you use the pill for the reason(s) stated above?

Years

| Cancer and Women’s Reproductive Health Study, Version 2 1^st^ December, 2013 Page \| 5 |
| --- |

1. For how many years in total have you used the pill (for any other purpose and as a family planning method).

Years

*(Add together the time you took the pill for other purpose indicated in question 8 above to the time used as a family planning method. Please write “0” if you used the pill for less than a year in total)*

1. Have you ever used DMPA? *(depot medroxyprogesterone acetate, a 3 monthly injection, also known as depo-provera)*

No *If* ***No,*** *please go to question 11*

Yes

If **YES**:

-about how old were you when you started using DMPA?  years.

-are you still on DMPA?  No, stopped – If so, when?  years ago

Yes, still using

-for how many years in total did you use DMPA?  Years

*(Add together the years and months when you actually were on DMPA.*

*Please write “0” if you used DMPA for less than a year in total)*

1. Have you ever used contraceptive implants? *(inserted below the skin on the upper arm)*

No *If* ***No,*** *please go to question 12*

Yes

| Cancer and Women’s Reproductive Health Study, Version 2 1^st^ December, 2013 Page \| 6 |
| --- |

If **YES**:

-about how old were you when you first had a contraceptive implant inserted?  years.

-are you still on a contraceptive implant?

No, stopped – If so, when?  years ago

Yes, still using

-for how many years in total did you use this method of contraception?  Years

*(Add together the years and months when you had a contraceptive implant. Please write “0” if you used contraceptive implants for less than a year in total)*

*-*what type of implants have you ever used?

Norplant

Jadelle

Implanon

Zarin

Other (*specify)*

I don’t know the type of implant I used

Cancer and Women’s Reproductive Health Study, Version 2 1^st^ December, 2013 Page | 7

1. Have you ever used an intra-uterine contraceptive device? *(IUCDs/coil/loop)*

No *If* ***No,*** *please go to question 13*

Yes If **YES**:

-about how old were you when you first had an IUCD inserted? years.

-are you still using an IUCD?  No, stopped – If so, when? years ago

Yes, still using

-for how many years in total did you use an IUCD?  years

*(Add together the years and months when you used an IUCD.*

*Please write “0” if you used IUCDs for less than a year in total)*

*-* What type of IUCD(s) have you used?

Copper T/copper 7

Multiload

Mirena

Other (*specify)*

I don’t know the type of IUCD I used.

1. How many sexual partners have you had in your life?

Partners

1. Have you ever had a sexual partner who has had a vasectomy?

No *If* ***No,*** *please go to question 15*

Yes

| Cancer and Women’s Reproductive Health Study, Version 2 1^st^ December, 2013 Page \| 8 |
| --- |

If **YES**:

-for how many years in total have you relied on vasectomy for family planning

Years

*(Add together the years and months you relied on vasectomy for family planning. Please write “0” if the total time is less than a year.)*

1. Have you ever used male or female condoms as a family planning method?

No *If* ***No,*** *please go to question 16*

Yes

If **YES**:

-for how many years in total did you use this method of contraception?  Years

1. Have you ever used any other type of contraceptive not included in the above questions?

No *If* ***No,*** *please go to question 17*

Yes – **If yes**, please fill in the following details:

| **Type of contraceptive** | **Your age when you started using it** | **Your age when you stopped using it** *(put “0” if you are still using it)* | **Years in total that you have been on this contraceptive** *(put ‘’0” if less than one year)* |
| --- | --- | --- | --- |
|  |  |  |  |
|  |  |  |  |
|  |  |  |  |
|  |  |  |  |

| Cancer and Women’s Reproductive Health Study, Version 2 1^st^ December, 2013 Page \| 9 |
| --- |

**SECTION C: QUESTIONS ABOUT YOU AND YOUR FAMILY**

1. Have your periods **NOW** stopped?

*Cross “****No****” – if you are still having regular periods now, even if they are because you are taking HRT.*

*Cross* ***“irregular****” – if your periods have been irregular and you think it might be because of the menopause*

*Cross “****Yes”*** *– if you are not having periods now, either because of your menopause, after hysterectomy (removal of the womb) or after stopping HRT.*

**No**

**Irregular**

**Yes**- If **Yes**, how old were you when they **stopped**?  Years

1. Other than the times you were on hormonal contraceptives, would you describe your periods as regular or irregular?

Regular *(number of days between one period and the next is almost fixed, variations of two to three days allowed)*

Irregular *(number of days between one period and the next varies*)

1. What is/was the average number of days between your periods? (*this is from the first day after your period to the last day of your next period)*

Days

1. How many children have you had? *(please include stillbirths*)  Children

| Cancer and Women’s Reproductive Health Study, Version 2 1^st^ December, 2013 Page \| 10 |
| --- |

1. When was each child born, and for how many months did you breastfeed each child, if at all?

| **BIRTH ORDER** | **DATE OF BIRTH**  (if you had twins or triplets please repeat the same date for each child) | | | **BREASTFEEDING**  *(months that you breastfed each child; put “0” if you did not breastfeed that child and “1” if you breastfed for one month or less*) |
| --- | --- | --- | --- | --- |
|  | Day | Month | Year |  |
| 1^st^ child | / | / |  | months |
| 2^nd^child | / | / |  | months |
| 3^rd^ child | / | / |  | months |
| 4^th^ child | / | / |  | months |
| 5^th^ child | / | / |  | months |
| 6^th^ child | / | / |  | months |
| 7^th^ child | / | / |  | months |
| 8^th^ child | / | / |  | months |
| 9^th^ child | / | / |  | months |
| 10^th^ child | / | / |  | months |

1. Have you had any spontaneous miscarriages or induced abortions?

No

Yes If **Yes**, how many?  Abortions/ Miscarriages.

| Cancer and Women’s Reproductive Health Study, Version 2 1^st^ December, 2013 Page \| 11 |
| --- |

1. Has any member of your family been diagnosed with cancer?

No

Yes

*If* ***YES****, please fill in the following details*

| How are you related *(e.g this person is my uncle, aunt, niece, cousin)* | Type of cancer *(e.g. prostate, colon,stomach)* | How old was she/he when the diagnosis was made? (*give age in years)* |
| --- | --- | --- |
|  |  |  |
|  |  |  |
|  |  |  |
|  |  |  |
|  |  |  |

**SECTION D: QUESTIONS ABOUT YOUR HEALTH**

1. Have you had a hysterectomy (your womb removed)?

No

Yes – **If yes**, how old were you?  Years

1. Have you had **ONE** ovary removed?

No

Yes – **If yes**, how old were you?  Years

1. Have you had **BOTH** ovaries removed?

No

Yes – **If yes**, how old were you?  Years

1. Have you been sterilised *(had your tubes tied/clipped)*?

No *If* ***No,*** *please go to question 28*

Yes

| Cancer and Women’s Reproductive Health Study, Version 2 1^st^ December, 2013 Page \| 12 |
| --- |

**If yes:**

-how old were you when you were sterilised?  Years

Has the sterilisation been reversed?

No

Yes

**If yes:** - how old were you when it was reversed?  Years

1. Have you ever been diagnosed with any type of cancer?

No

Yes

*If* ***YES****, please fill in the following details*

| Type of cancer *(for example, ovary, womb/uterus, bowel, breast, etc)* | Your age when the diagnosis was first made *(give age in years)* |
| --- | --- |
|  |  |
|  |  |
|  |  |
|  |  |

1. Have you been told by your doctor that you have:

|  | No | Yes | Age first diagnosed |
| --- | --- | --- | --- |
| Uterine fibroids? |  |  | years old |
| Endometriosis? |  |  | years old |
| Benign ovarian cysts? |  |  | years old |
| Infertility? |  |  | years old |

| Cancer and Women’s Reproductive Health Study, Version 2 1^st^ December, 2013 Page \| 13 |
| --- |

1. Have you ever used drugs that promote ovulation to overcome difficulty conceiving a child?

No

Yes

If **YES**, what is/was the total length of time you used it?

Years and  Months

1. Have you ever used HRT (hormone replacement therapy, also known as post-menopausal hormone)?

No

Yes – **If yes**, how many years in total?  total years of use

*(Please put “0” if you used/have used HRT for less than one year in total)*

| Cancer and Women’s Reproductive Health Study, Version 2 1^st^ December, 2013 Page \| 14 |
| --- |

**SECTION E: MORE QUESTIONS ABOUT YOURSELF**

1. What is your highest secondary school qualification?

None

NZ School Certificate in one or more subjects *or*

National Certificate Level 1 *or*

NCEA level 1

NZ Sixth Form Certificate in one or more subjects *or*

National Certificate level 2 *or*

NZ UE before 1986 in one or more subjects *or*

NCEA level 2

NZ Higher School Certificate *or*

Higher Leaving Certificate

NZ University Bursary/Scholarship *or*

National Certificate level 3 *or*

NCEA level 3 *or*

NZ Scholarship

Other secondary school qualification **gained in NZ**. Print what it is:

Or  other secondary school qualification **gained overseas**.

1. Apart from secondary school qualifications, do you have another completed qualification?

(*Don’t count qualifications that take less than 3 months of full-time study to get*)

No

Yes

If **YES**, print your highest qualification and the main subject; for example:

Qualification: TRADE CERTIFICATE

Subject: ELECTRICAL ENGINEERING

| Cancer and Women’s Reproductive Health Study, Version 2 1^st^ December, 2013 Page \| 15 |
| --- |

Qualification (and level, if applicable)

Subject

1. What is your main occupation? (for example PRIMARY SCHOOL TEACHER, CLOTHING MACHINIST, MOTEL MANAGER, RECEPTIONIST etc. *Please list if you have more than one main occupation*)


1. If you are retired, what was your main occupation before retirement (*Please list if you had more than one main occupation*)?


| Cancer and Women’s Reproductive Health Study, Version 2 1^st^ December, 2013 Page \| 16 |
| --- |

1. From all your sources of income, what will the total income be :

- That you yourself got
- Before tax or anything was taken out of it
- In the 12 months ending 31^st^ March this year?

Loss

Zero income

$1-$5,000

$5,001-$10,000

$10,001-$15,000

$15,001-$20,000

$20,001-$25,000

$25,001-$30,000

$30,001-$35,000

$35,001-$40,000

$40,001-$50,000

$50,001-$60,000

$60,001-$70,000

$70,001-$100,000

$100,001-$150,000

$150,001 or more

Cancer and Women’s Reproductive Health Study, Version 2 1^st^ December, 2013 Page | 21

| Cancer and Women’s Reproductive Health Study, Version 2 1^st^ December, 2013 Page \| 17 |
| --- |

1. In the last 10 years, about how much wine, beer or spirits did you usually drink a week? (*Please cross one box for each type*)

| **Wine** | **Lager/Cider/Beer** | **Spirits** |
| --- | --- | --- |
| (glasses per week) | (half pints per week) | (tots per week) |
| none | none | none |
| less than 1 | less than 1 | less than 1 |
| 1-3 | 1-3 | 1-3 |
| 4-6 | 4-6 | 4-6 |
| 7-10 | 7-10 | 7-10 |
| 11-15 | 11-15 | 11-15 |
| 16-20 | 16-20 | 16-20 |
| 21+ | 21+ | 21+ |

If you drink **wine** is it

Mostly red mostly white

About the same amount of red and white

1. Have you ever been a regular smoker of one or more cigarettes a day?

No *If* ***No,*** *please go to question 42*

Yes

1. How old were you when you started smoking regularly?

Years old

1. Are you a smoker now?

No If **No**,how old were you when you stopped smoking?  Years old

Yes

| Cancer and Women’s Reproductive Health Study, Version 2 1^st^ December, 2013 Page \| 18 |
| --- |

1. About how many cigarettes do you/did you smoke on average each day? *(if you are an ex-smoker, how many did you smoke on average when you smoked?)*

Cigarettes per day

1. Have you ever applied/used talcum powder on your groin area?

No

Yes – **if yes**, how many years in total?  total years of use

*(Please put “0” if you used/have used talcum powder for less than one year in total)*

1. How tall are you? *(please give to the nearest centimetre/inch)*

Centimetres. *Or* Feet and Inches

1. About how much do you weigh now?

Kilograms. *Or*  Pounds

1. What did you weigh at 18 years of age?

Kilograms. *Or* Pounds

1. What has been your usual weight after 18 years of age excluding the times when you were pregnant?

Kilograms. *Or* Pounds

1. On which date did you fill in this questionnaire?

//*(Fill as day/month/year)*

| Cancer and Women’s Reproductive Health Study, Version 2 1^st^ December, 2013 Page \| 19 |
| --- |

1. We may wish to contact you for further information about this study. If you are agreeable to this, please indicate your telephone contact below.

Telephone number: Area code (_ _ _ _)

Phone number (_ _ _ _ _ _ _ _ _ _ _)

Mobile phone number: _______________________________________

What is the best time to call you? _______________________________________

1. If you would like to receive a summary of the results of this study, please provide an address or other way for us to send it to you at the end of the study.

|  |
| --- |
|  |
|  |

*Thank you for taking time to fill in this questionnaire. Please post it back to us together with the consent form in the envelope provided.*

| Cancer and Women’s Reproductive Health Study, Version 2 1^st^ December, 2013 Page \| 20 |
| --- |
